# Intentional rounding: a realist evaluation using case studies in acute and care of older people hospital wards

**DOI:** 10.1186/s12913-023-10358-1

**Published:** 2023-12-02

**Authors:** Mary Leamy, Sarah Sims, Ros Levenson, Nigel Davies, Sally Brearley, Stephen Gourlay, Giampiero Favato, Fiona Ross, Ruth Harris

**Affiliations:** 1 Florence Nightingale Faculty of Nursing, Midwifery and Palliative Care, King’s College London, James Clerk Maxwell Building, 57 Waterloo Road, London, SE1 8WA UK; 2 Independent researcher, London, UK; 3 College of Health, Medicine and Life Sciences, Brunel University London, Uxbridge, UK; 4 Faculty of Health, Science, Social Care and Education, Kingston University, London, UK; 5 Kingston Business School, Kingston University, London, UK

**Keywords:** Intentional rounding, Nursing care delivery, Patient safety, Fundamental nursing care, Compassionate care, Checklist, Realist evaluation, Realist synthesis

## Abstract

**Background:**

In response to concerns about high hospital mortality rates, patient and carer complaints, a Mid Staffordshire NHS Foundation Trust public inquiry was conducted at the request of the UK government. This inquiry found serious failures in the quality of basic care provided and as a consequence, recommended that patients should have more regular visits, organised at predictable times from nursing staff. Intentional rounding, also known as nursing ward rounds, was widely adopted to meet this need.

**Objective:**

To test, refine or refute eight programme theories to understand what works, for whom, and in what circumstances.

**Setting:**

Six wards (older people and acute wards) in three NHS trusts in England.

**Participants:**

Board level and senior nursing managers (*N* = 17), nursing ward staff (*N* = 33), allied health and medical professionals (*N* = 26), patients (*N* = 34) and relatives (*N* = 28) participated in an individual, in-depth interview using the realist method. In addition, ward-based nurses (*N* = 39) were shadowed whilst they conduced intentional rounds (240 rounds in total) and the direct care of patients (188 h of patient care in total) was observed.

**Methods:**

The mixed methods design included: Phase (1) Theory development - A realist synthesis was undertaken to identify any programme theories which were tested, refined and/or refuted, using data from phases 2 and 3; Phase (2) A survey of all English NHS acute Trusts; Phase (3) Six case studies of wards involving realist interviews, shadowing and non-participant observations, analysis of ward outcome and cost data; and Phase (4) Synthesis of findings from phases 1, 2 and 3.

**Results:**

The realist synthesis identified eight programme theories of intentional rounding: ‘Consistency and comprehensiveness’, ‘Accountability’, ‘Visibility of nurses’, ‘Anticipation’, ‘Allocated time to care’, ‘Nurse-patient relationships’, ‘Multi-disciplinary teamwork and communication’ and ‘Patient empowerment’. Key findings showed that of the original eight programme theories of intentional rounding, only *two* partially explained how the intervention worked (‘Consistency and comprehensiveness’ and ‘Accountability’). Of the remaining six programme theories, the evidence for two was inconclusive (‘Visibility of nurses’ and ‘Anticipation’) and there was no evidence for four (‘Allocated time to care’; ‘Nurse-patient relationships’; ‘Multi-disciplinary teamwork and communication’; and ‘Patient empowerment’).

**Conclusions:**

This first theory-informed evaluation of intentional rounding, demonstrates that the effectiveness of intentional rounding in the English healthcare context is very weak. Furthermore, the evidence collected in this study has challenged and refuted some of the underlying assumptions about how intentional rounding works. This study has demonstrated the crucial role context plays in determining the effectiveness of an intervention and how caution is needed when implementing interventions developed for the health system of one country into another.

**Supplementary Information:**

The online version contains supplementary material available at 10.1186/s12913-023-10358-1.

## Background

 In England, the introduction of intentional rounding was in response to the findings from a public inquiry, known as the Francis Inquiry, which pinpointed serious errors in patient care at the Mid Staffordshire NHS Trust. The inquiry produced two reports, one covering the patient neglect and poor standards of care and the other focussing upon the inadequacy of regulatory and supervisory systems, negative organisational culture, acceptance of low standards and poor  management and leadership. One of the recommendations, entitled “Communication with and about patients” (Recommendation 238), was that ‘*regular interaction and engagement between nurses and patients and those close to them should be systematised through regular ward rounds’* [1], p1700).

Such an approach to providing nursing ward rounds, known as intentional rounding, had been developed in the US [2], by a private, for-profit healthcare consultancy firm. During intentional rounding, nurses use a standardised checklist and documentation to check every patient on a ward, usually once an hour. This checklist includes an opening and closing phrase; then questions about four patient care areas, known as the ‘4 P’s’ (Positioning, Personal needs, Pain and Placement of items); then a review of the care environment; checking if the patient needs anything; letting the patient know the time the nurse will be back; and completing the paperwork to record that the round has been completed [2, 3]. As the Studer Group state in their intentional rounding toolkit:

“*The purpose of rounding on patients is to demonstrate our organization’s commitment to providing quality care to our patients and families – and to validate that this level of care is occurring with every patient, every time… When done consistently, leaders can manage the patient expectations and experiences proactively as opposed to finding out later that a gap occurred, perhaps through complaint letters, poor patient outcomes, or poor patient experience”* [4], p4).

Some authors [5] have reported that rounding was a means of ensuring quality and safe healthcare, and improving levels of consumer satisfaction [2, 6]. An integrative review of the available evidence concluded that intentional rounding has positive outcomes on patient satisfaction and safety [7]. The review highlighted that many of the included studies had weakness in design and cautioned about how this would have an impact on the credibility of review findings. The authors noted that the effectiveness of intentional rounding is influenced by external factors, such as leadership, training on intentional rounding, workload, staffing levels and experience level and ward layout [7]. All of these issues have relevance today with the continuing pressures on maintaining workforce, morale and retention.

The first known introduction of mandatory rounding was reported by Sheedy, [8], but is usually credited to the Studer Group [9]. Sheedy described how their institution responded to the increased levels of patient acuity and workload for qualified nurses by introducing unit hostesses (who were described as unlicenced staff members). Hostesses were not care givers, but were there to satisfy patient’s basic needs such as answering call lights and responding to other nonmedical or non-nursing requests. In the US, intentional rounding was introduced as a response to hospital funding eligibility requirements, to raise patient experience scores.

Widespread adoption of intentional rounding occurred in England between 2011 and 2014, as an immediate UK government response to public and professional safety and quality concerns about patient care was required to convince the public that the failings identified at Mid Staffordshire NHS Trust were being taken seriously. All Directors of Nursing were instructed by David Cameron, the UK Prime Minister that they should ‘*Comply or explain*’ why they were not implementing intentional rounding [10]. This was interpreted by many Directors of Nursing in NHS hospitals that introducing intentional rounding was mandatory [10]. In addition to the UK and USA, intentional rounding has been implemented in Australia and Iran [5, 11–13].

Realist evaluation is a theory-driven form of research, underpinned by the philosophical position of critical realism. Realist evaluation assumes that programmes are complex, with different components, and when they are introduced into social systems, they may or may not work as intended. In other words, we know that because people are different in terms of their culture, gender, class, beliefs, individual capacities and so on, the way they think about and respond to the resources offered by programmes differs, which contributes to whether programmes such as intentional rounding are successful in achieving their expected outcomes. According to realist evaluators, programmes will only work effectively when the underlying causal mechanisms which lead to the desired outcomes are actively switched on by the right circumstances [14]. The purpose of the current study was to test, refine and/or refute programme theories of what works, for whom, in what circumstances and why, as developed in an earlier phase of the study, a realist synthesis of the literature [15]. This realist synthesis highlighted the previous lack of theoretical causal explanations of intentional rounding, the many ambiguities surrounding its purpose and limited evidence of how it works in practice. The original mechanisms identified in the realist synthesis are reported in Table 1.

**Table 1:** Hypothesised mechanisms of intentional rounding (Stage 1) (Table reproduced from [15]

Mechanism title	Mechanism (Resources)	Mechanism (Reasoning/Responses)
M1: Consistency and comprehensiveness^a^	Intentional rounding helps keep patient care consistent through the use of a structured, systematic approach, ensuring all patient needs are met and potentially less obvious aspects of care are considered and managed at every round. Intentional rounding also helps ensure that family members are provided with consistent care and information in line with their needs (e.g. the need for information, to be respected and to be comforted). It can also prompt agency staff to deliver care to a required standard.	This provides reassurance and confidence in the quality of care to patients, their family members and staff.
M2: Allocated time^a^	Intentional rounding gives nurses allocated ‘time to care’ (i.e., time to check that patients are comfortable and their needs are being met, thereby treating patients with dignity and replaces ‘presumed care’).	This helps nurses to organise their work and feel able to prioritise this aspect of nursing care.
M3: Accountability^a^	Staff are required to complete and sign the intentional rounding record to say they have carried out hourly checks.	This makes staff feel personally accountable for the standard of care. This enables ward managers to monitor and audit the standard of care provided by nursing staff.
M4: Nurse-patient relationships and communication^a^	Intentional rounding provides increased and improved communication between staff, patients and family members and ensures that the patients’ perceived basic fundamental needs are met. It also provides more opportunities for positive nurse-patient relationships to develop based upon trust, respect and caring.	This enables staff to get to know patients better and become more aware of their needs, notice unusual behaviours/appearances and detect subtle/significant changes that can impact upon comfort and safety.
M5: Visibility^a^	Intentional rounding increases the visibility/presence of nurses within a unit by increasing the time that nurses spend in the direct vicinity of their patients (i.e., it gets nurses to the patient’s bedside).	This relieves the uncertainty and anxiety often associated with vulnerable patients’ hospital experience (i.e., the inability to predict when care will be delivered and when someone will be available to assist them with care). This is comforting to family members because it denotes frequent and continuous assessment of the patient and their needs.
M6: Anticipation^a^	Intentional rounding enables nurses to anticipate/pre-empt and proactively address patient needs instead of being reactive and waiting for patient call bells and alarms.	This ensures that all patients receive regular care instead of unequally distributed care amongst patients focussed towards those who have frequent call bell use.
M7: Staff communication and/or teamworking	Intentional rounding provides healthcare professionals with documented evidence.	This is used to enhance staff communication, teamwork and prioritise care in future rounds.
M8: Patient empowerment	Intentional rounding provides an opportunity for nursing staff, patients and family members to get to know each other better.	This empowers patients to ask for what they need in order to maintain their comfort and wellbeing.

NB – All of the mechanisms below were identified in empirical research papers and six (marked^a^) were also identified in the grey and policy literature

## Methods

Between 2014 and 2019, we undertook a realist evaluation of intentional rounding. This paper provides only an overview of the study methods; more details have been published elsewhere [10, 15, 16]. In this paper, we extend the analysis and interpretation of how contextual barriers and enabling factors impacted upon the underlying causal mechanisms determining how intentional rounding works and discuss wider implications for nursing practice in England and internationally.

### Realist synthesis

As this was a large realist evaluation study, a realist synthesis methodology was the most appropriate means of generating initial programme theories [17]. The review involved three stages of searching the literature, followed by a stakeholder consultation event [10, 15]. The review was conducted in line with realist synthesis guidance, so rather than critically appraising the quality of studies, papers were judged against a ‘fitness for purpose’ criteria, where both relevance and rigour were taken into consideration [18, 19].

### National survey

In 2015, in a national, cross-sectional survey of all NHS acute Trusts in England [10], we sought to understand the way in which context influenced the implementation of intentional rounding^1^. Survey questions captured details such as when intentional rounding was introduced; what staff training and engagement activities for intentional rounding were organised prior to implementation; frequency and timing of rounds; and how rounds were documented and audited. A high response rate was achieved. Of the 155 English NHS hospital Trusts that were sent a survey, 108 (70%) responded. One hundred and five (97%) trusts reported that they had partially implemented intentional rounding, but there was considerable variability in exactly how and when they had done it.

### Case studies

Three NHS acute Trusts were purposively sampled from this national survey to comprise our case study sites, using characteristics such as geographical location, hospital size and ward layout, to ensure the sites were as diverse as possible. During the process of recruiting our three case study sites, two other Trusts were initially invited, but declined to participate; one due to the need to focus on special measures set by the CQC and the other due to recent changes in senior nursing leadership.

In each case study hospital site, two wards were identified: one acute care ward and one care of older people ward (reflecting the specific types of wards of greatest concern in the Francis Inquiry). Each hospital site had different ward layouts. For example, Site One had four to six-bedded bays, Site Two was predominately single rooms whilst in Site Three, one ward was a mix of four to six-bedded bays and single rooms whilst another was a nightingale ward^2^. Trust documentation and senior manager interview data were used to provide individual ward profiles and to describe how intentional rounding was implemented, developed, and supported (see Figure S1: Summary table of ward profile data). The research team spent between two to three weeks on each ward, taking it in turns to work nursing day and night shifts to observe nurses and patients. To profile each case study ward, routine data was collected (e.g., Human Resources data on training, staffing and sickness levels, vacancy rates, and data which could characterise the ward, for example ward layout, types of nursing shifts, and intentional rounding documentation such as records and policies).

Across all case study sites, senior managers (*N* = 17), ward-based nurses (*N* = 33), allied health and medical professionals (*N* = 26), patients (*N* = 34) and relatives (*N* = 28) participated in individual, realist interviews. The interview schedules were designed to enable the research team to test, refine and refute the programme theories developed in the realist synthesis. Two approaches to non-participant observation were conducted. Firstly, ward-based nurses were ‘shadowed’ to explore how they carried out rounds (*N* = 39) and how they fitted intentional rounding in with other care delivery activities. Nursing staff were purposively sampled to attain a range of grades and levels of experience and all provided informed consent. Researchers took narrative field notes of what they observed, which were used to complete two protocols: one to record individual rounding interactions, and the other to assess fidelity to the original rounding intervention, as set out by the Studer Group. Secondly, researchers ‘shadowed’ patients over a 2–4 h period to observe individual interactions between patients and nurses so that all types of nursing interactions could be described and to see how intentional rounding contributed to overall care received. Overall, a total of 240 rounds were observed within 188 h of care delivery observation. Observation fieldnotes and interview transcripts were managed using NVivo, and analysed thematically using framework analysis [20] to identify staff, patient and carer experiences and contextual variation. Data were analysed concurrent to data collection, to identify causal explanations for how intentional rounding works (i.e., Context-Mechanism-Outcome configurations (CMOs) [14]. RAMESES reporting guidelines were adhered to throughout [21].

## Results

### Revised evidence-informed programme theory

**Fig. 1:**
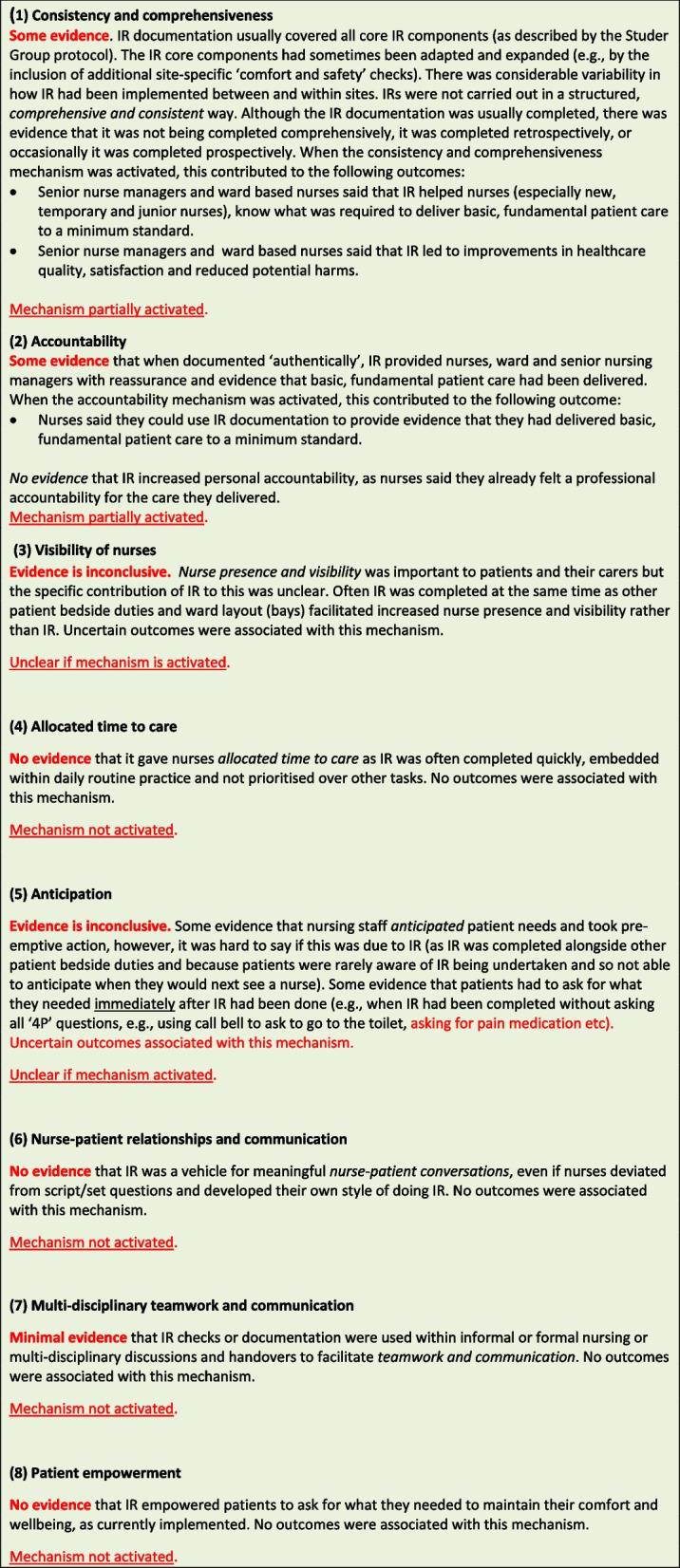
Revised evidence-informed programme theory

 Data synthesis highlighted that two of the original eight mechanisms were partially activated^3^. These were the ‘Consistency and comprehensiveness’ mechanism and the ‘Accountability’ mechanism. Evidence for a further two mechanisms – ‘Visibility of nurses’ (see Figure S2) and ‘Anticipation’ (see Figure S3) – remained inconclusive. The remaining four mechanisms – ‘Allocated time to care’ (see Figure S4); ‘Nurse-patient relationships’ (see Figure S5); ‘Multidisciplinary teamwork and communication (see Figure S6); and ‘Patient empowerment’ (see Figure S7) were not activated and had minimal supporting evidence. The revised evidence-informed programme theory is presented in Fig. 1. The observation of intentional rounding in practice, to look at fidelity to the original intervention, is highlighted in Table 2.

**Table 2:** Observations of intentional rounding in practice: Fidelity to the original intervention (i.e., Studer Group protocol) Table has been reproduced [10]

	Opening phrase where nurse introduces self	Positioning (check comfortable, assess pressure sore risk)	Personal needs (assess personal needs, assist with toileting)	Pain (ask patient to rate pain on a scale of 0–10)	Placement (ensure any items are in easy reach)	Environment (assess care environment e.g., temperature of room, fall hazards etc.)	Closing phrase (e.g., Is there anything else I can do for you before I go?”	Patient informed of when the nurse will return	Documents round *immediately after* interaction	Documents round *retrospectively* (within observation period)	Documents round *prospectively* (i.e., before talking to patient)	Doesn’t document *at all* (within observation period)
Total observations in all three case study sites												
**Fully observed**	39/240 (16%)	48/240 (20%)	54/240 (23%)	15/240 (6%)	51/240 (21%)	13/240 (5%)	6/240 (3%)	2/240 (0.8%)	**181/240 (75%)**	21/240 (9%)	4/240 ( 2%)	31/240 (13%)
** Partially observed**	87/240 (36%)	16/240 (7%)	8/240 (3%)	48/240 (20%)	3/240 (1%)	7/240 (3%)	15/240 (6%)	7/240 (3%)				
** Observed in any form (full/partial)**	**126/240 (53%)**	64/240 (27%)	62/240 (26%)	63/240 (26%)	54/240 (23%)	20/240 (8%)	21/240 (9%)	9/240 (4%)	**206/240 (86%)**			
** Not observed**	113/240 (47%)	**171/240 (71%)**	**173/240 (72%)**	**173/240 (72%)**	**183/240 (76%)**	**215/240 (90%)**	**217/240 (90%)**	**229/240 (95%)**				
** Unable to observe**	1/240 (0.4%)	5/240 (2%)	5/240 (2%)	4/240 (2%)	3/240 (1%)	5/240 (2%)	2/240 (0.8%)	2/240 (0.8%)	2/240 (0.8%)	1/240 (0.4%)		
**Site One**												
** Fully observed**	4/89 (4%)	13/89 (15%)	11/89 (12%)	5/89 (6%)	14/89 (16%)	4/89 (4%)	1/89 (1%)	1/89 (1%)	76/89 (85%)	5/89 (6%)	1/89 (1%)	7/89 (8%)
** Partially observed**	20/89 (22%)	4/89 (4%)	1/89 (1%)	14/89 (16%)	1/89 (1%)	3/89 (3%)	1/89 (1%)	0/89 (0%)				
** Observed in any form (full/partial)**	24/89 (27%)	17/89 (19%)	12/89 (13%)	19/89 (21%)	15/89 (17%)	7/89 (8%)	2/89 (2%)	1/89 (1%)	**82/89 (92%)**			
** Not observed**	**65/89 (73%)**	**70/89 (79%)**	**75/89 (84%)**	**68/89 (76%)**	**74/89 (83%)**	**80/89 (90%)**	**86/89 (97%)**	**87/89 (98%)**				
** Unable to observe**	0/89 (0%)	2/89 (2%)	2/89 (2%)	2/89 (2%)	0/89 (0%)	2/89 (2%)	1/89 (1%) (1%)	1/89 (1)	0/89	0/89		
**Site Two**												
** Fully observed**	19/108 (18%)	27/108 (25%)	25/108 (23%)	6/108 (6%)	26/108 (24%)	5/108 (5%)	5/108 (5%)	0/108 (0%)	83/108 (77%)	9/108 (8%)	3/108 (3%)	11/108 (10%)
** Partially observed**	51/108 (47%)	7/108 (6%)	4/108 (4%)	26/108 (24%)	2/108 (2%)	2/108 (2%)	12/108 (11%)	3/108 (3%)				
** Observed in any form (full/partial)**	**70/180 (65%)**	34/108 (31%)	29/108 (27%)	32/108 (30%)	28/108 (26%)	7/108 (6%)	17/108 (16%)	3/108 (3%)	**95/108 (88%)**			
** Not observed**	37/108 (34%)	**72/108 (67%)**	**77/108 (71%)**	**74/108 (69%)**	**79/10 (73%)**	**100/10 (93%)**	**90/108 (83%)**	**104/108 (96%)**				
** Unable to observe**	1/108 (1%)	2/108 (2%)	2/108 (2%)	2/108 (2%)	1/108 (1%)	1/108 (1%)	1/108 (1%)	1/108 (1%)	1/108 (1%)	1/108 (1%)		
**Site Three**												
** Fully observed**	16/43 (37%)	8/43 (18%)	18/43 (42%)	4/43 (9%)	11/43 (26%)	4/43 (9%)	0/43 (0%)^a^	1/43 (2%)	22/43 (51%)	7/43 (16%)	0/43 (0%)	13/43 (30%)
** Partially observed**	16/43 (37%)	5/43 (12%)	3/43 (7%)	8/43 (19%)	0/43 (0%)	2/43 (5%)	2/43 (5%)	4/43 (9%)				
** Observed in any form (full/partial)**	**32/43 (74%)**	13/43 (30%)	**21/43 (49%)**	12/32 (28%)	11/43 (26%)	6/43 (14%)	2/43 (5%)	5/43 (12%)	**29/43 (67%)**			
** Not observed**	11/43 (26%)	**29/43 (67%)**	**21/43 (49%)**	**31/43 (72%)**	**30/43 (70%)**	**35/43 (81%)**	**41/43 (95%)**	**38/43 (88%)**				
** Unable to observe**	0/43 (0%)	1/43 (2%)	1/43 (2%)	0/43 (0%)	2/43 (5%)	2/43 (5%)	0/43 (0%)	0/43 (0%)	1/43 (2%)	0/43 (1%)		

^a^Case Study Site 3’s IR documentation did not require staff to ask the question, “Is there anything else I can do for you?”

### CMO 1: consistency and comprehensiveness – partially activated

**Fig. 2:**
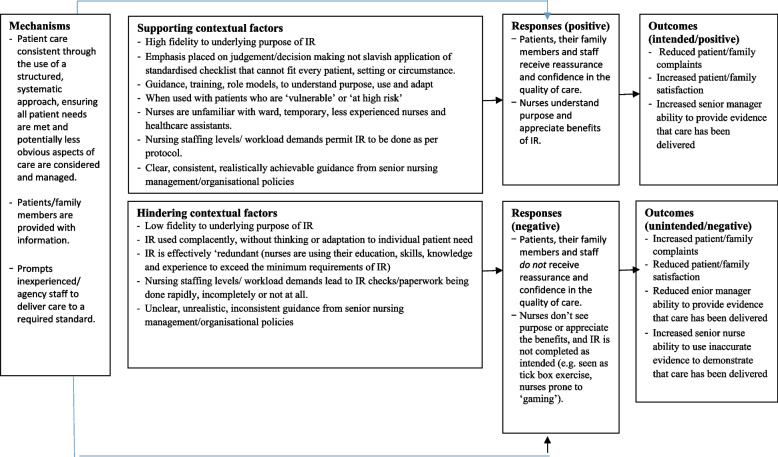
Consistency and comprehensiveness: specific contextual factors that hinder or enable the mechanism

Figure 2 illustrates the specific contextual factors that hindered or enabled the ‘Consistency and Comprehensiveness’ mechanism.

Many patients and carers commented about their experiences of consistency and comprehensiveness of care. The majority who raised this issue found rounding to be beneficial for general observations, and for specific issues such as hydration checks. A positive outcome of regular, consistent, and comprehensive patient rounds was increased reassurance for carers that their loved ones were being cared for. Nursing opinion was divided on whether rounding should be in place for all patients. Those in favour said that rounding all patients meant that, “*no one and nothing gets missed*”, which addressed a key concern raised by the Francis Inquiry. As one interviewee stated:

*“Sometimes… you’ll be assessing them at a level that actually is probably a bit of overkill, but you will know that everybody got it*” (Senior nursing manager).

Those against rounding for all patients felt that it was more beneficial for some (e.g., older, bed-bound patients and those with dementia or pressure ulcers etc.) than for others (e.g., younger, more mobile, independent patients). Nevertheless, no ward nurses said that rounding should be applied in a structured, standardised way at *every* round and our observations confirmed that this was the case. Instead, staff talked about the need to have flexibility in their approach and to be able to use their clinical judgement/common sense to tailor their rounds according to the needs of each patient. Indeed, senior nurses expressed concerns that rounds might be done in a rigid, scripted, task-orientated way as it was felt that this would compromise holistic care. Nurses said that the question, ‘*Is there anything else I can do for you*?’ was often asked more casually, through phrases such as “*Do you need anything?”* or *“Can I help you?”.* Some nurses said they chose not to ask patients this question at all because they did not have the capacity to respond to their answer. This may explain why this question was only asked in 9% of all our observations of rounds.

Senior nurses, ward based nurses and medical and allied healthcare staff variously described intentional rounding as a *“checklist” or “aide memoire”*, which was thought to support standardised care and clinical governance through checking and documenting the delivery of care. Both ward based and senior nurses felt that having visual reminders in the form of a checklist was particularly helpful for bank, or newly qualified/recruited nurses to alert them to safety risks, such as pressure risk assessment, food and fluid intake or falls risk assessment. However, the original US model of intentional rounding and its ‘4P’s’ is not a long list. In our interviews, many of the checklist items that were considered to be the most useful part of intentional rounding were *not* part of the original US model, but were local adaptations (e.g., food and fluid charts, body map, hearing aids and dentures etc.). This variation was also found in the national survey where many trusts said they had adapted the intervention to include a wide range of additional items and assessments [10]. Nurses spoke of the difficulties associated with adding more elements to what was originally intended to be a quick check. Checklists are frequently used in the aviation industry and in healthcare (for example, the World Health Organisation Surgical Safety Checklist [22] to ensure consistency and comprehensiveness and to improve safety. Such checklists are usually most effective when time is not critical, when the series of tasks are too lengthy to be committed to memory or when there are advantages to standardising performance [23]. Our data suggested that these advantages are only partially achieved through the use of checklists in hospital ward settings.

### CMO 3: accountability – partially activated

**Fig. 3:**
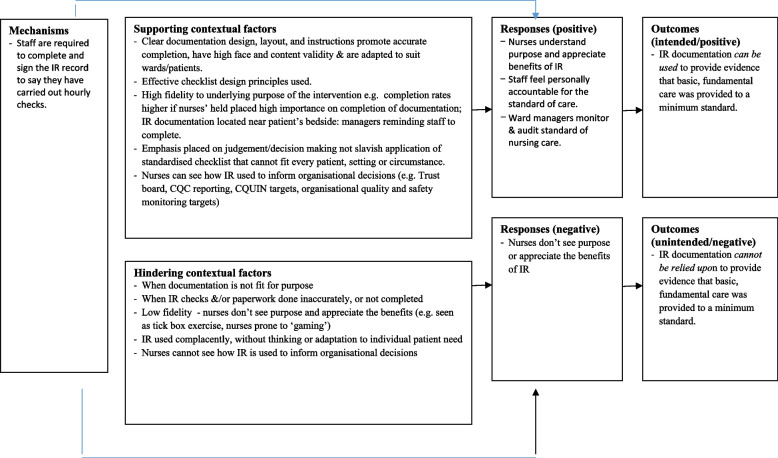
Accountability: specific contextual factors that hinder or enable the mechanisms

Figure 3. illustrates the specific contextual factors that hinder or enable the “Accountability” mechanism.

This mechanism was not apparent to patients and their family members, though it resonated strongly with members of staff. All senior nursing staff (*N* = 17) and half of ward based nurses (*N* = 16/33) stated that the main benefit of intentional rounding was the documented evidence that it provided:

“… *from an executive nurse’s perspective, gives me some assurance that if you’ve ticked box then you’ve done it, and if not ticked box, you haven’t.”* (Senior nursing manager).

Many repeated a particular phrase: “*If you haven’t written it down, you haven’t done it”*, which emphasises how entrenched the need to evidence that care has been delivered is in nurses’ way of thinking. Several participants talked of rounding as a ‘safety net’ in a target-driven, risk averse and litigious health system, which is increasingly financially challenged and struggles with staff shortages. One senior nurse said that it would take a brave person to stop doing intentional rounding. Although rounding was no longer thought to be required or audited by the Care Quality Commission (CQC - the independent regulator of health and social care in England), there was a perception that if something went wrong, the CQC would state that rounding should have been in place. Similarly, there was a consensus amongst the medical and allied healthcare professionals interviewed that rounding documentation was valued because it provided evidence of the delivery of nursing care, should that be required in case of patient complaints or for coroners’ inquests. In practice, data collected from our interviews, nurse shadowing and patient observations revealed that rounding documentation provided flawed and unreliable evidence, in respect to its accuracy, trustworthiness, completeness and meaningfulness:

“*….from what I see on an audit, it literally is a tick, tick, tick. Now, for me, that doesn’t necessarily mean it was done.*” (Matron).

Six ward based nurses reported that they, or their colleagues, had either completed the documentation without undertaking the round or had completed the documentation before undertaking the round. During our fieldwork observations, we witnessed such examples of prospective completion of documentation. This involved either writing all the times of interaction onto the form in advance and then filling the rest in later or writing the incorrect time of interaction onto the form, in order to fit within the allocated hourly slot. The temptation to document rounds prospectively, or without completing the round, was exacerbated by senior management pressure to ensure that documentation was always up to date. At one site, staff said they appreciated being told by management that they needed to be honest on their rounding documentation and complete it for the exact time at which the round had taken place. They felt that inaccuracies on the documentation had decreased following this guidance. They also felt it important for their Trust to see an accurate picture of how regularly they could interact with patients, so that they were aware of the reality of their busy workloads. Some interviewees spoke of the dangers of rounding becoming a ‘tick box exercise’ and highlighted the balance between making it feasible to do yet detailed enough to be meaningful.

One medical consultant felt that the danger of focussing only upon task completion was that changes in patient conditions may not be detected over the longer term. Some senior nurses also expressed concern that the principle of regularly checking patients was being lost in the focus on completion of documentation. Some ward based nursing staff felt that whilst rounding may improve standards in hospitals that were providing poor care, they did not see how it could benefit hospitals that were already rated highly. Several participants talked about the influence of the Francis Inquiry on nursing and senior nurse managers from all sites talked about their aspirations to develop nursing further. In two sites, senior nurse managers hoped that rounding would one day not be needed and that the approach to regular, individualised contact with patients would be the cultural norm for nursing. This was expressed as a desire for nursing to evolve and become accountable for outcomes rather than processes:

*“…I guess, for me, Francis is old now and we need to, as a profession, be evolving in, and measuring ourselves and deciding going forward, how do we demonstrate good quality care, what is an outcome that we want to see, rather than a measure that we want to implement that would give us an outcome.”* (Senior nursing manager).

### CMO 5: visibility of nurses - inconclusive

Figure S2 illustrates the specific contextual factors that hinder or enable the “Visibility of nurses” mechanism.

One of the initial programme theories for intentional rounding was that it enhanced nurse visibility by increasing the amount of time nurses spent in close proximity to their patients. Many of the patients and carers interviewed provided positive comments about nurse visibility or presence on the ward, while eight made negative comments. However, these comments were about the visibility of staff in general and not specifically about the visibility associated with rounding. Findings from our staff interviews and observations suggested that the ward layout was highly influential in determining whether rounding increased nurse visibility. Half of the ward based nurses interviewed (*N* = 15) stated that rounding was better at increasing nurse visibility in single room wards rather than bay-based settings. However, there was some evidence in our observations that rounding did not increase nurse visibility in single room settings. For example, in Site 2 (where wards consisted of all single rooms), staff were told to carry out rounding alongside other tasks. As nurses were already in the room with patients completing other tasks, rounding did not increase the frequency of direct interaction between nurses and patients.

### CMO 6: anticipation - inconclusive

Figure S3 illustrates the specific contextual factors that hinder or enable the “Anticipation” mechanism.

There was insufficient evidence that rounding enables nurses to anticipate and proactively address patient needs rather than reacting to or waiting for patient call bells. Frontline nurses said they felt that anticipating patients’ needs was something they would do with or without rounding. Observations also demonstrated little other evidence of the anticipation mechanism in action. Nursing staff never gave patients a specific time that they would return to them, either because they were not aware of the importance of this or because they were worried they would not return on time. Thus, the anticipation mechanism could not be triggered. Similarly, because patients were not told that rounding was happening, they could not anticipate that nurses would return. Instead, nursing staff were observed to tell their patients to use the call bell whenever they needed anything.

### CMO 2: allocated time to care – not activated

Figure S4 illustrates the specific contextual factors that hinder or enable the “Allocated time to care” mechanism.

It was the absence of this mechanism that was discussed by the majority of ward based nurses (*N* = 25) and senior nurses (*N* = 11). Senior nurses were aware of the time constraints that nurses experienced in delivering care, particularly when wards were short-staffed, and understood how a perceived lack of time influenced the way rounding was conducted:

“’*I won’t ask the questions or I won’t look at them, because they’re going to ask me and I haven’t got time to answer those questions. They might ask me, I might have to go and find someone else and I haven’t got time to do it’, I’ve heard that, staff say that.*” (Senior nursing manager).

Staff usually multi-tasked, combining rounding with other activities and they were frequently interrupted as they did so, which led to some staff being observed documenting their rounds retrospectively. This suggests that nurses did not have sufficient time to do rounding as required, as they had to prioritise other activities. None of the medical and allied healthcare staff thought intentional rounding provided nurses with allocated time to care and indeed two participants thought it would involve extra work for nurses. There were no patient or family carer comments relating to this mechanism, although some patients and carers reported having to wait to receive care.

### CMO 4: nurse-patient relationships and communication – not activated

Figure S5  illustrates the specific contextual factors that hinder or enable the “Nurse-patient relationships and communication” mechanism.

There was minimal evidence that rounding provided greater opportunity for positive nurse–patient relationships to develop. Four senior nurses thought that rounding had no influence on nurses’ communication with patients, saying it simply reduced their interactions to a list of tasks to complete:

*“… the contact becomes transactional rather than enriching, so you’re not having a conversation with that patient”* (Senior nursing manager).

Few ward based nurses talked of the impact rounding could have upon nurse-patient relationships, and while a third said it increased the *frequency* of their communication with patients, these communications were generally seen as brief. For example, nurses described just ‘popping in’ to check that patients were okay rather than having lengthy discussions with them. Some staff felt that brief, but more frequent communication was appreciated by patients and their carers, giving them confidence that they would not be forgotten. However, not all outcomes of more frequent communication were positive. For example, some said that patients (particularly those who had been on the ward for longer periods of time) found the increased frequency of communication to be a nuisance. A further unanticipated negative outcome was that rounding could raise patients’ and their family carers’ expectations of how much time nursing staff should be spending with them – an expectation that nurses felt they were not able to achieve.

Nurses were often observed talking with patients; however, the majority of these interactions were not as part of an intentional round. However, it is possible that any information discussed or observed by nurses during these conversations may have contributed in some way to the completion of the rounding documentation. When rounds were specifically observed, sometimes the focus was on the content of the round and communication between patient and nurses was very short (e.g., with nurses asking if the patient was OK or had any pain etc.). Longer conversations tended to occur when patients were having a wash or other procedure. Some staff were observed having general social chats with patients, whilst at other times staff were observed to be less chatty and to be focused on care delivery. On several occasions, nursing staff were observed to complete the rounding documentation when patients were either asleep or away from their bed. Some staff were even observed to complete the rounding documentation without communicating with patients who were awake and next to them. Many comments were made about communication and relationships with staff by both patients and their family carers but it was rarely possible to specifically link these comments to intentional rounding.

### CMO 7: Teamwork and communication – not activated

Figure S6 illustrates the specific contextual factors that hinder or enable the “Teamwork and communication” mechanism.

Our interviews revealed that prior awareness amongst medical and allied healthcare staff about intentional rounding was low or non-existent. Whilst there was some overlap between rounding questions and the kinds of questions medical and allied healthcare staff asked, they said they would ask the nurse or patient directly, rather than referring to the rounding paperwork. Most senior nurses (*N* = 12) said they thought intentional rounding facilitated some communication between nurses and healthcare assistants (HCAs), although this tended to focus on whether patients had been checked, rather than upon sharing information about patients or facilitating the way staff worked together.

Similarly, our observations did not demonstrate intentional rounding to be particularly beneficial for team communication or teamworking. Nursing staff were regularly observed catching up with each other during shifts, but intentional rounding was rarely mentioned at these times. Furthermore, whilst all sites had a bedside handover, rounding was rarely discussed at these times. Where intentional rounding was discussed in relation to managing work, it often appeared to be about task allocation (e.g., how to allocate rounds between staff). Once again. no comments were made on this mechanism by patients or family carers.

#### CMO 8: patient empowerment – not activated

Figure S7 illustrates the specific contextual factors that hinder or enable the “Patient empowerment” mechanism.

Four ward based nurses thought that because intentional rounding enabled nursing staff to go to the patient’s bedside more regularly, patients therefore became more familiar and confident with the nursing staff and more likely to ask for what they needed. However, the same number of staff disagreed with this and felt that rounding made no difference to patient empowerment. Only one of the senior nurses thought that rounding had the potential to empower patients if conducted in the right way. Overall, there was little evidence of patients being empowered by intentional rounding, though observers acknowledged that this would be difficult to see. On one occasion, a nurse was observed to ask a patient about their pain during an intentional round and this seemed to assist the patient to ask about his other needs. However, both patients and their family carers were observed to ask nursing staff questions throughout the day, calling them over when required, rather than waiting until rounds took place. In sum, intentional rounding was not found to be a vehicle for patient empowerment as the process was rarely explained to or detectable by, patients.

## Discussion

This study is the first to test and refine the intentional rounding programme theories previously made explicit in a realist synthesis of the literature, which set out eight programme theories about what works, for whom, and in what circumstances [15]. The interview and observational findings reveal only two of the eight mechanisms proposed to explain the impact of intentional rounding were enabled within the care delivery context, and even then, only partially. There is inconclusive or no evidence for the remaining mechanisms. We now discuss these findings by placing them within the wider context of the nursing profession and NHS and offer some reflections about why only two of the eight underlying mechanisms were activated.

### Intentional rounding adds to the tension inherent in the delivery of systematised care vs. individual patient care

The unique contribution of nursing to patient care, experience and outcomes has been the subject of considerable discussion and debate for a long time. What is rarely disputed is the importance of the relationship between the patient and the nurse in the delivery of therapeutic care. However, tension can arise between task oriented approaches to fundamental care delivery and the need to develop and maintain the relational aspects of providing patient care [24].

There is a danger that systematised care in the form of tasks, with the mechanistic connotations and insinuation of being a chore, risks devaluing and lowering the visibility of the knowledge and skill that the provision of fundamental care requires. The systematised, structured approach to intentional rounding was seen to emphasise transactional care delivery rather than relational, individualised patient care. As such there seemed to be perverse consequences of a tool which was intended to reduce risk by overseeing care in a systematic way, which may indeed prevent nurses paying attention to individual patient need or engaging in meaningful interaction. This tension is summed up by one senior nurse manager, who said: *“… I don’t know if it* [intentional rounding] *fits what we do and I don’t know if it helps what we do or it becomes more of a hindrance.”* A nursing routine which involves regular and repetitive use of rounding checklists of all patients, day and night and using checklists that are often far longer than the original ‘4 P’s’ rounding list is onerous. One nurse in our study commented that *‘if I did intentional rounding as it should be done, I would have no time in the day to do anything else’*. As previously mentioned, in other countries the rounding task has been delegated to others. For example, it has been reported [25] that the success of rounding was often heavily reliant on the Emergency Department Assistant, who took on a key role in undertaking rounding. However, when the assistant was absent from the department, implementing rounding became difficult. The flexibility with which rounding was observed to be delivered in this study was potentially a way for nurses to manage this tension by allowing them to individualise patient care to some extent, while still being compliant to organisational expectations.

### The influence of the international healthcare organisation context on intentional rounding

There are several key differences in the political and commercial context of healthcare organisations in the UK and US that influence variation in outcomes of intentional rounding. These differences relate to: (1) the characteristics of the healthcare settings, (2) the purpose behind introducing intentional rounding and the way it was implemented, and (3) the need for an intervention to improve standards of fundamental care on individual wards and hospital settings.

Firstly, the specific intervention recognised as intentional rounding was developed in the US. Unlike the healthcare system in the UK, which is paid for via taxation, the US healthcare system is largely owned by private sector businesses, with quality of care determined by how much patients have to pay. In the US, patients are often in single rooms whereas in the UK, there are fewer single rooms and wards are more open plan, with a ‘Nightingale’ or bay layout design, so nurses are naturally more visible to patients. Secondly, in the UK, intentional rounding was introduced rapidly and offered as an emergency solution to address failures in the quality of patient care. Whilst there was an evaluation study of intentional rounding in a couple of demonstrator sites, this was conducted at the same time that it was extensively implemented in the UK [26]. In hindsight, the widespread implementation in the UK was done with insufficient understanding about how and why it should work, or indeed without compelling evidence that it did work in the US [27]. In addition, there was no central or external guidance or support about how to implement it, or indeed financial resources and instead Trusts were encouraged to find their own ways of implementing it. This contrasts with other interventions which were transferred from a US to UK setting, such as Schwartz Rounds, which have been implemented with a high degree of fidelity [28]. Thirdly, intentional rounding offers the means to achieve a minimum standard of fundamental patient care, and we found some evidence that rounding was believed to be valued in hospitals settings where basic procedures for providing safe, satisfactory levels of care were required.

### Organisational culture and the management of risk

A strong theme within the data was that nursing staff highly valued the evidence intentional rounding provided to demonstrate care had been delivered. This reassurance was an important outcome of intentional rounding for nursing staff working within NHS organisational cultures which are preoccupied by risk avoidance and management. Nurses also felt anxious at times because they were unable to conduct the rounds as often as their Trust required or complete the documentation immediately after the round due to being interrupted or other time pressures. They rationalised not undertaking the rounds as per Trust policy because they already knew their patients’ needs having spent time with them undertaking other care activities. Bed occupancy was more than 92% in each case study site which is far higher the recommended rate of 85% (http://nhsproviders.org/news-blogs/news/bed-occupancy-rateshit-record-high). The findings of this study show uncertainty about the accuracy of the intentional rounding documentation. Nurses were observed to carry out intentional rounding activities without completing the documentation and also to complete the rounding documentation without carrying out the nursing care activities required. Therefore, the intentional rounding documentation is arguably an unreliable source of evidence of the delivery or non-delivery of nursing care. Senior nursing managers recognised this challenge and were reticent about the accuracy of intentional rounding as a record of care delivery. However, it was clear it reassured them that some care had been delivered. Some senior nursing managers acknowledged the use of rounding as a safety-net or as a defence against complaints of poor practice at an individual nurse, ward, and organisational level. They saw intentional rounding as a minimum standard rather than something to aspire to and talked about their ambition to develop and innovate nursing practice beyond the confines of intentional rounding., However, they felt restricted by a culture of compliance and risk aversion perpetuated by the performance management approach prevalent in the NHS that inhibited innovation [29]. One senior manager commented that stopping intentional rounding would be a *‘brave’* thing to do. Previous research studies have recognised the impact of organisational risk management on practice. Others recognise the importance of having reporting systems and a ‘no blame’ culture in place to enable and support openness and ‘learning from mistakes’ [e.g., [30–32]]. There is a danger that intentional rounding inadvertently encourages defensive and risk averse practice, but arguably diminishes nursing. The focus on documentation may be good for ensuring an audit trail, but potentially risks nurses not feeling supported to be proactive and enquiring or engaging in the worlds of patients and their preferences and concerns. Most nurses emphasised the documentation aspect of intentional rounding demonstrating that their actions were influenced by the dominant NHS organisational culture focused on risk mitigation rather than relationship-based care.

Our study is the first to use realist evaluation to understand the mechanisms at play in this complex intervention and this is an important strength. It captured the perceptions and experiences of key stakeholders providing a comprehensive view of intentional rounding from those leading, implementing, delivering, and receiving it. The research team spent two to three weeks on each case study ward to focus the evaluation on how intentional rounding worked ‘on the ground’. Direct observation of care delivery has enabled us to challenge many of the assumptions reported in the international research literature about how intentional rounding works. Following interviews with senior nurse managers, we were often asked to interview other senior nurses because they had an important perspective to contribute, or that they would find it useful to talk with us. Therefore more managers were interviewed than planned which increased the richness of the data on the perspectives of nursing leaders and executives, often a hard to reach group [33].

Limitations of the study included that it was sometimes difficult to see when intentional rounding was happening. This was because it was often delivered flexibly and at the same time as other care. Researchers were careful not to interrupt nurses during observations, so asking questions to seek clarification was kept to a minimum. If we had interviewed nursing staff straightaway after shadowing them, we would have been able to ask them about how and why they were delivering care in the way they did and the contribution of rounding to patient care. Furthermore, nurses provided care for the same patients during a shift and often for several days at a time and may have got to know their needs and preferences without needing to frequently ask, which is likely to have influenced how they interacted with patients. This knowledge may have influenced how nurses delivered intentional rounding although it is not possible for researchers to observe this. Furthermore, case study wards included in the research were selected by senior nursing managers at the Trusts, which may have introduced bias. We also identified some disparities between data collected from the different methods used (e.g., what staff talked about in the interviews and what researchers observed) [34]. The potential explanations for these disparities are highlighted in Table 3.

**Table 3:** Potential explanations for disparities between quantitative and qualitative findings

Approaches to explore data disparities [34]	Application to our evaluation (qualitative = case study interviews/observations)
(i )Treating methods as fundamentally different	Quantitative and qualitative components had different (although related) research questions, and approaches to data collection/analyses are based on fundamentally different theoretical paradigms. While this may partially explain the disparities, the quantitative data recording observations of the individual components of IR related to whether there was fidelity to the original IR intervention when we shadowed selected nurses and when we observed individual patients over the duration of a nursing shift, both day and night. Put simply, did we observe IR taking place and if so, to what extent did it fit with the original IR intervention design. Qualitative: The purpose of the semi-structured interviews was broader, to understand test, refine and refute the eight programme theories from the realist review of the literature.
(ii) Exploring the methodological rigour of each component	Both individual interviews and observations were conducted rigorously. Quantitative: The non-participatory observations through nurse shadowing and individual patients were extensive. Ward-based nurses (*N*=39) were shadowed, leading to us observing 240 rounds, and the direct care of patients (188 hours of patient care in total) was observed. We also examined whether the IR documentation was completed, following our observations. Qualitative: The interview sample was diverse and included Board level and senior nursing managers (*N*=17), nursing ward staff (*N*=33), allied health and medical professionals (*N*=26), patients (*N*=34) and relatives (*N*=28). We actively sought disconfirming cases. ‘Thinking aloud’ interviews with nursing staff during shadowing would have been beneficial in helping us understand why they did not conduct an IR fully or at all, when they were scheduled.
(iii) Exploring data set comparability	Broadly, the data sets were comparable, as all interviews and observations occurred in the same case study sites and the quota sample of numbers of patients, carers, nurses, senior nurses, and allied health professionals were achieved at each site. There were differences between the case study sites though, in terms of geographical location, hospital size, ward layout and length of time running IR.
(iv) Collection of additional data and making further comparisons	Using data from both qualitative and quantitative sources provides a more comprehensive picture of IR in each of the three case study sites, including whether the different contexts supported the intervention or not. The research timetable did not allow for analysis to inform further data collection in the qualitative components.
(v) Exploring whether or not the intervention under study worked as expected	Quantitative: The observational data was key to drawing conclusions around whether the intervention worked as expected. We consistently found that IR could not be observed in any form (with individual components being 76-98% not observed (site 1), 34- 96% not observed (site 2) and 26-95% not observed (site 3). This happened despite the ward staff being aware they were being observed as part of a research study on IR. Qualitative: Interviewees emphasised the value of IR in relation to accountability and consistency and comprehensiveness, predominately through having IR documentation that the rounds had taken place.
(vi) Exploring whether or not the outcomes of the quantitative and qualitative components match	Interviewees often spoke about their hopes and potential for IR, whereas the observational data revealed what was occurring in actual practice. Quantitative: The key finding can be summarised as IR was not being delivered with high fidelity to the original IR, and/or was frequently not observed to be occurring at all. Qualitative: In relation to the question of fidelity and whether IR was being delivered, interviewees agreed that time constraints influenced how IR was being delivered and that they were encouraged to multi-task and combine IR with other activities.

## Conclusions

This first national theory-informed evaluation of intentional rounding, demonstrates that the effectiveness of intentional rounding in the English healthcare context is very weak. This is a significant contribution to understanding of how nursing care is delivered in the NHS. The theoretical framework underpinning the evaluation was developed from the international literature and this, together with the rich description of healthcare context, enables the study to inform intentional rounding in other international contexts. Although intentional rounding did offer some perceived benefits, it was seen as a reductive, formulaic process that did little to support the individual patient/nurse relationship. It thus devalues nursing as an ally of the individual patient and their needs and preferences. It is heartening that nurses expressed scepticism of intentional rounding and were honest about its shortcomings.

Both the contextual circumstances and reasons for the development and adoption of intentional rounding in the US are different from those in the UK. After widespread implementation and adaptation of intentional rounding procedures to suit a UK context, our evidence suggests that it is not achieving the intended outcomes. Although, perhaps in a way, intentional rounding did fulfil one purpose of its introduction: to provide an immediate, politically expedient, solution to reassure the public as the Francis Inquiry report was published. One recommendation could be to consider abandoning intentional rounding and replacing it with nursing procedures which are fit for purpose and for a UK setting. However, the situation is more complex than this. Nurses and senior nurse managers may be reluctant to relinquish the reassurance and protection the intentional rounding documentation gave them in the current NHS culture. Furthermore, some of the contextual factors that have inhibited intentional rounding are highly likely to influence any alternative interventions should intentional rounding be de-implemented [35]. Therefore, we recommend that the findings of this study inform national nursing conversations about the use of intentional rounding in the UK and internationally. This would help hospitals and senior nursing leaders decide how to respond to this new evidence and whether there are alternative interventions which support fundamental nursing care delivery for patients.

### Supplementary Information

**Additional file 1: Figure S1.** Summary table of ward profile data.

**Additional file 2: Figure S2.** Visibility: specific contextual factors that hinder or enable the mechanisms to fire.

**Additional file 3: Figure S3.** Anticipation: specific contextual factors that hinder or enable the mechanisms to fire.

**Additional file 4: Figure S4.** Allocated time: specific contextual factors that hinder or enable the mechanisms to fire.

**Additional file 5: Figure S5.** Nurse–patient relationships and communication: specific contextual factors that hinder or enable the mechanisms to fire.

**Additional file 6: Figure S6.** Multi-disciplinary communication &/or teamworking: specific contextual factors that hinder or enable the mechanisms to fire.

**Additional file 7: Figure S7.** Patient empowerment: specific contextual factors that hinder or enable the mechanisms to fire.

## Data Availability

The datasets generated during the current study are available from the corresponding author on reasonable request.

## Abbreviations

4P’s: Intentional rounding checklist of positioning, personal needs, pain and placement of items
CMO: Context-mechanism-outcome configuration
CQC: Care Quality Commission
CQUIN: Commissioning for Quality and Innovation
HCA: Healthcare assistant
IR: Intentional rounding
NHS: National Health Service
UK: United Kingdom
US: United States of America

## Footnotes

1 In realist research, context is defined as including the individual characteristics of key stakeholders (e.g., ward based nurses, nursing managers, patients), the interpersonal relationships required to support an intervention; the organisational setting (e.g., leadership, culture and norms, governance arrangements); and the wider (infra) structure (e.g., the availability of funding, resources, competing priorities of the NHS).

2 Nightingale ward: A type of hospital ward that consists of one large room without subdivisions, and 24 to 34 beds arranged along. the sides.

3 ‘Mechanisms’ are referred to by realist researchers as being activated (or enabled to work), but also can be thought of as a light switch, which can be switched on or off, or perhaps dimmed (partially activated).
