# Supplementary material for: Intentional rounding: a realist evaluation using case studies in acute and care of older people hospital wards
Source: BMC Health Serv Res. 2023 Dec 2;23:1341. doi: 10.1186/s12913-023-10358-1 (PMC10693126; doi:10.1186/s12913-023-10358-1)
Supplement: Supplementary file 1 — Additional file 1: Figure S1. Summary table of ward profile data. [file 12913_2023_10358_MOESM1_ESM.docx]

**Figure S1.Summary table of ward profile data**

|  | Site 1 | | Site 2 | | Site 3 | |
| --- | --- | --- | --- | --- | --- | --- |
| Trust |  | |  | |  | |
| *Size* | Large (>800 beds) | | Large (>800 beds) | | Very Large (>1000 beds) | |
| *Location* | Urban | | Urban with rural catchment area | | Urban | |
| *Bed occupancy* | >94% | | >95% | | >92% | |
| Ward |  |  |  |  |  |  |
|  | ***Ward a*** | ***Ward b*** | ***Ward a*** | ***Ward b*** | ***Ward a*** | ***Ward b*** |
| *Specialty* | Health care for Older People | Acute medicine, endocrinology | Acute trauma orthopaedic | Health care for Older People | Acute medicine, cardiac and respiratory | Health care for Older People |
| *No. of beds* | 24 | 24 | 32 | 32 | 26 | 18 |
| *Predominant ward layout* | 3-7 bed bays | 3-7 bed bays | Single, en-suite rooms | Single, en-suite rooms | 4 bed bays | Nightingale |
| *Nursing organisation* |  | Three teams |  | Four teams |  |  |
| *Shift pattern* | 12-hour shifts | 12-hour shifts | Combination of 12-hour shifts and early (0700-1330) and late (1300-1930) shifts | Combination of 12-hour shifts and early (0700-1330) and late (1300-1930) shifts | 12-hour shifts | 12-hour shifts |

| Nursing Team |  |  |  |  |  |  |
| --- | --- | --- | --- | --- | --- | --- |
| Nursing staff Establishment at the time of data collection (WTE) | February 2017 | January 2017 | April 2017 | May 2017 | July 2017 | July 2017 |
| *Band 7* | 1.0 | 1.0 | 1.0 | 1.0 | 1.0 | 1.0 |
| *Band 6* | 2.0 | 2.0 | 3.6 | 3.6 | 4.9 | 2.1 |
| *Band 5* | 15.19 | 17.29 | 17.2 | 20.9 | 21.5 | 12.7 |
| *Band 4* | 9 | 0 | 2.6 | 6.6 | 0 | 0 |
| Band 3 | 0 | 0 | 6.5 | 5.8 | 0 | 0 |
| Band 2 | 15.09 | 11.09 | 19.6 | 7.1 | 9.5 | 12.4 |
| Total | 33.28 | 31.38 | 50.5 | 45.0 | 36.9 | 28.2 |
| *Vacancy rate (FTE)* | 3 RN vacancies  1 HCA vacancy | 4 RN vacancies  0 HCA vacancy | 4.4 | 8.1 | 19.78% | 9.69% |
| *Agency/bank use* | 127 shifts covered by temporary staff | 82 shifts covered by temporary staff | 6.7 (FTE)  2.0 RN, 4.7 HCA | 11.4 (FTE)  4.0 RN, 7.4 HCA | 8.06 (FTE) | 4.57 (WTE) |
| *Sickness* | RN – 19.74%  HCA – 0% | RN – 11.56%  HCA – 45.08% | - 1. (FTE)   3.0% | 2.2 (FTW)  5.4% | Not available - tbc | Not available – tbc |
|  | **Site 1** | | **Site 2** | | **Site 3** | |

| Implementation of IR |  |  |  |
| --- | --- | --- | --- |
| *When introduced* | Some discrepancy, sometime between 2009 and 2012, most likely 2011 | Some discrepancy, sometime between 2014-2016, most likely 2014 | Due to staff changes, exact date is unclear, around 2012/2013 |
| *Circumstances* | Part of a strategy to reduce patient harm and increase care quality | Part of the strategy to reduce increased falls risk as a result move to predominantly single room environment and develop compassionate care | Ward managers able to decide whether to implement IR or not, the majority of wards were thought to be implementing it at the time of the study. |
| *Staff involvement* | Not initially, but recognised that this will ill-judged and staff were involved to re-design which was piloted on some wards before rollout to all wards | Managers reported a period of testing and piloting although think IR was probably implemented too quickly across the Trust. |  |
| *Documentation* | Four page A4 booklet. Has been frequently revised according to perceived need. Includes 4Ps questions and ‘Is there anything else I can do for you?’ question. | Two sided form. Includes 4Ps questions and ‘Is there anything else I can do for you?’ question. | Two versions of the IR form depending on pressure sore risk score (Waterlow score< 10 and ≥10). Both are part of 49 page nursing documentation bundle. IR form for Waterlow score <10  Includes 4Ps questions but **not** the ‘Is there anything else I can do for you?’ question. |
| *Trust IR policy* | Detailed trust policy. | Detailed Trust standard operating procedure | No formal IR policy |
| *Frequency of IR* | Hourly between 0800 and 2200, two hourly between 2200 and 0800 hours. Time intervals pre-written onto form. | Frequency could vary according to risk assessment as long as rationale was recorded. Minimum frequency of four hourly. Time of IR not specified so specific time was entered by staff. | IR form for Waterlow score <10 is two hourly with time intervals written onto form.  IR form for Waterlow score ≥10 asks staff to ‘*continuously*’ complete the form and staff are required to write in time the patient is seen. |
| *Who does IR* | Both HCA and RN. RN required to complete IR at 0200, 0800, 1200, 1600, 2000 hours | Any member of clinical staff who had read the IR standard operating procedure and had received training in SKIN and Falls bundles. | Both RN and HCA staff. |
| *Adaptation of IR beyond Studer format* | IR documentation included questions about mobility, bed rail position, special mattress, body map to record skin integrity and presence of medical devices | IR documentation included questions offering drinks/snacks, falls prevention, body map to record skin integrity and presence of medical devices. Space available to document any actions resulting from IR | IR form for Waterlow score ≥10 included assessing skin inspection, nutrition, special mattress needs. |
