# Supplementary material for: Intentional rounding: a realist evaluation using case studies in acute and care of older people hospital wards
Source: BMC Health Serv Res. 2023 Dec 2;23:1341. doi: 10.1186/s12913-023-10358-1 (PMC10693126; doi:10.1186/s12913-023-10358-1)
Supplement: Supplementary file 2 — Additional file 2: Figure S2. Visibility: specific contextual factors that hinder or enable the mechanisms to fire. [file 12913_2023_10358_MOESM2_ESM.docx]

**Figure S2. Visibility: specific contextual factors that hinder or enable the mechanisms to fire**

**Mechanisms (Resources)**

- Increases the visibility/presence of

nurses within a unit

by increasing the time that nurses spend in the direct vicinity of their patients (ie, it gets nurses to the patient’s bedside).

**Responses (positive)**

- Nurse visibility relieves patients’ uncertainty/ anxiety
- Family members who see frequent/ continuous assessment of the patient.

**Supporting contextual factors**

- Single room ward layout – higher contribution of IR towards increasing visibility of nurses.
- Nurses complete IR checks at patient bedside (e.g. IR documentation kept at end of bed/in patient’s room)

**Outcomes (intended/positive)**

- Reduced patient/family complaints
- Increased patient/family satisfaction

**Responses (negative)**

- Fails to relieves patients’ uncertainty/ anxiety
- Comforting to family members who see frequent/ continuous assessment of the patient.

**Outcomes (unintended/negative)**

- Increased patient/family complaints
- Reduced patient/family satisfaction

**Hindering contextual factors**

- Bay or Nightingale ward layout - lower contribution of IR towards increasing visibility of nurses
- Nurses complete IR checks at a distance (e.g. IR documentation can be completed away from patient’s bedside/completed via mobile computer pad)
