# Supplementary material for: Intentional rounding: a realist evaluation using case studies in acute and care of older people hospital wards
Source: BMC Health Serv Res. 2023 Dec 2;23:1341. doi: 10.1186/s12913-023-10358-1 (PMC10693126; doi:10.1186/s12913-023-10358-1)
Supplement: Supplementary file 3 — Additional file 3: Figure S3. Anticipation: specific contextual factors that hinder or enable the mechanisms to fire. [file 12913_2023_10358_MOESM3_ESM.docx]

**Figure S3. Anticipation: specific contextual factors that hinder or enable the mechanisms to fire**

**Outcomes (intended/positive)**

- All patients receive regular care instead of unequally distributed care among patients, focused towards those who have frequent call bell use.

**Responses (positive)**

- Nurses respond to patient’s needs during IR check

**Supporting contextual factors**

- IR check completed at patient’s bedside, as per protocol, including telling patient when nurse will return
- High fidelity to underlying purpose of IR
- Patient need can be anticipated and patients are able to communicate their needs

**Mechanisms**

**(Resources)**

- IR enables nurses to anticipate/

pre-empt & proactively address patient needs instead of being reactive and waiting for patient call bells & alarms.

**Outcomes (unintended/negative)**

- Patients use the call bell or call out to attract nurses’ attention to ensure their needs are met

**Responses (negative)**

- Nurses *do not respond* to patient’s needs during IR check

**Hindering contextual factors**

- Nurses complete IR checks at a distance (e.g. IR documentation can be completed away from patient’s bedside/completed via mobile computer pad)
- IR checks do not involve any interaction with patients (i.e. documentation completed silently so IR process is invisible to patients/family)
- Patient needs cannot be anticipated &/or patient cannot communicate them
