# Supplementary material for: Intentional rounding: a realist evaluation using case studies in acute and care of older people hospital wards
Source: BMC Health Serv Res. 2023 Dec 2;23:1341. doi: 10.1186/s12913-023-10358-1 (PMC10693126; doi:10.1186/s12913-023-10358-1)
Supplement: Supplementary file 4 — Additional file 4: Figure S4. Allocated time: specific contextual factors that hinder or enable the mechanisms to fire. [file 12913_2023_10358_MOESM4_ESM.docx]

**Figure S4. Allocated time: specific contextual factors that hinder or enable the mechanisms to fire**

**Outcomes (intended/positive)**

- IR can be completed and done as per protocol
- Reduced patient/family complaints
- Increased patient/family satisfaction
- Increased senior manager ability to provide evidence that care has been delivered

**Supporting contextual factors**

- Higher nursing staffing levels/lower workload demands
- Senior staff encourage and legitimise nurses to prioritise IR

**Responses (positive)**

- Nurses can organise their work/ feel able to prioritise this aspect of nursing care

**Mechanisms**

**(Resources)**

- Gives nurses allocated ‘time to care’ (ie, time to check that patients are comfortable, needs are being met, thereby treating patients with dignity.
- Replaces ‘presumed care’.

**Outcomes (unintended/negative)**

- IR being done rapidly, incompletely or not at all.
- Increased patient/family complaints
- Decreased patient/family satisfaction
- Reduced senior manager ability to provide evidence that care has been delivered

**Responses (negative)**

- Nurses *cannot* organise their work/ feel able to prioritise this aspect of nursing care

**Hindering contextual factors**

- Lower nursing staffing levels/ higher workload demands
- Senior staff encourage nurses to do IR at same time as performing other duties, rather than a discreet activity
