# Supplementary material for: Intentional rounding: a realist evaluation using case studies in acute and care of older people hospital wards
Source: BMC Health Serv Res. 2023 Dec 2;23:1341. doi: 10.1186/s12913-023-10358-1 (PMC10693126; doi:10.1186/s12913-023-10358-1)
Supplement: Supplementary file 5 — Additional file 5: Figure S5. Nurse–patient relationships and communication: specific contextual factors that hinder or enable the mechanisms to fire. [file 12913_2023_10358_MOESM5_ESM.docx]

**Figure S5. Nurse–patient relationships and communication: specific contextual factors that hinder or enable the mechanisms to fire**

**Mechanisms (Resources)**

- Provides increased and improved communication between staff, patients and family members,
- Ensures that patients’ perceived basic fundamental needs are met.
- Provides more opportunities for positive nurse– patient relationships to develop based on trust, respect and caring.

**Supporting contextual factors**

- Higher nursing staffing levels/lower workload demands
- High fidelity to underlying purpose of IR
- Nurses use IR as a vehicle for engaging patients in wider conversation and develop nurse-patient relationship

**Outcomes (intended/positive)**

- Increased interaction and improved nurse-patient relationships
- Increased opportunities to detect subtle/significant changes that can impact on comfort and safety

**Responses (positive)**

- IR provides opportunities for staff get to know patients better
- Staff become more aware of their needs, notice unusual behaviours/ appearances.

**Hindering contextual factors**

- Lower nursing staffing levels/ higher workload demands
- Low fidelity to underlying purpose of IR
- Nurses use script rigidly and only ask closed questions &/or complete IR checks silently

**Outcomes (unintended/negative)**

**Responses (negative)**

- Staff are able to get to know patients better
- Staff become more aware of their needs, notice unusual behaviours/ appearances.
