# Supplementary material for: Intentional rounding: a realist evaluation using case studies in acute and care of older people hospital wards
Source: BMC Health Serv Res. 2023 Dec 2;23:1341. doi: 10.1186/s12913-023-10358-1 (PMC10693126; doi:10.1186/s12913-023-10358-1)
Supplement: Supplementary file 6 — Additional file 6: Figure S6. Multi-disciplinary communication &/or teamworking: specific contextual factors that hinder or enable the mechanisms to fire. [file 12913_2023_10358_MOESM6_ESM.docx]

**Figure S6. Multi-disciplinary communication &/or teamworking: specific contextual factors that hinder or enable the mechanisms to fire**

**Outcomes (intended/positive)**

- Increased staff communication and teamwork

**Responses (positive)**

Staff use IR to enhance staff communication

and teamwork

Staff use IR to prioritise care.

**Supporting contextual factors**

- High fidelity to underlying purpose of IR
- Guidance, training, role models, to understand purpose, use and adapt when used with patients who are ‘vulnerable’ or ‘at high risk’
- Clear documentation design, layout, and instructions promote accurate completion, have high face and content validity & are adapted to suit wards/patients.

**Mechanisms**

**(Resources)**

- Provides healthcare professionals with documented evidence.

**Outcomes (unintended/negative)**

- No impact on staff communication and teamwork

**Responses (negative)**

Staff do not use IR to enhance staff communication

and teamwork

Staff *do not use* IR to prioritise care

**Hindering contextual factors**

- Low fidelity to underlying purpose of IR
- IR used complacently, without thinking or adaptation to individual patient need
- When documentation is not fit for purpose
