# Supplementary material for: Intentional rounding: a realist evaluation using case studies in acute and care of older people hospital wards
Source: BMC Health Serv Res. 2023 Dec 2;23:1341. doi: 10.1186/s12913-023-10358-1 (PMC10693126; doi:10.1186/s12913-023-10358-1)
Supplement: Supplementary file 7 — Additional file 7: Figure S7. Patient empowerment: specific contextual factors that hinder or enable the mechanisms to fire. [file 12913_2023_10358_MOESM7_ESM.docx]

**Figure S7. Patient empowerment: specific contextual factors that hinder or enable the mechanisms to fire**

**Outcomes (intended/positive)**

Increased patient comfort

Increased patient well-being

**Responses (positive)**

- Patients feel empowered and able to ask for what they need

**Supporting contextual factors**

- Patients and family *have some awareness* of IR, through having received an explanation about what it is and why it is being done
- High fidelity to underlying purpose of IR
- Nurses use IR as a vehicle for engaging patients in wider conversation and develop nurse-patient relationship
- Patients are told when nurses will be coming back to see them, as part of IR.

**Mechanisms**

**(Resources)**

- Frequent and regular contact provides an opportunity for nursing staff, patients and family members to get to know each other better.

**Outcomes (unintended/negative)**

Reduced patient comfort

Reduced patient well-being

**Responses (negative)**

- Patients do not feel empowered or able to ask for what they need.

**Hindering contextual factors**

- Patients and family *not aware of IR* (e.g. not told what IR is, or why being done)
- IR checks &/or paperwork done inaccurately, or not completed
- Nurses use script rigidly and/or only ask closed questions
- IR checks do not involve any interaction with patients (i.e. documentation completed silently so IR process is invisible to patients/family)
